# Matrine Attenuates D-Galactose-Induced Aging-Related Behavior in Mice *via* Inhibition of Cellular Senescence and Oxidative Stress

**DOI:** 10.1155/2018/7108604

**Published:** 2018-11-27

**Authors:** Kaiyue Sun, Pengyu Yang, Rong Zhao, Yuting Bai, Zijiao Guo

**Affiliations:** College of Animal Science and Veterinary Medicine, Shanxi Agricultural University, Taigu 030801 Shanxi, China

## Abstract

The present study was designed to evaluate the effects of matrine (MAT) on D-galactose- (D-gal-) induced aging and relative mechanism. Vitamin E at the dose of 100 mg/kg was used as a standard positive control. MAT significantly improved the D-gal-induced recognition and spatial memory impairment in novel object recognition and Y maze tests, and exercise endurance decreased in the weight-loaded swimming test at 2 and 10 mg/kg. We found that D-gal treatment induced noticeably aging-related changes such as reducing thymus coefficients, increasing the pathological injury and cellular senescence of liver, spleen, and hippocampus, as well as an increase in cyclin-dependent kinase inhibitor *p16*, *p19*, and *p21* gene expression and the interleukin*-1β* expression in the liver and hippocampus. MAT showed effective protection on such changes. Furthermore, MAT decreased the oxidative stress of the liver, plasma, and brain, as evidenced by increased total antioxidant capacity, total superoxide dismutase, and catalase activities and decreased the malondialdehyde level. Additionally, there was a significant positive correlation between swimming time in weight-loaded swimming time and thymus index. MAT ameliorated aging-related disorder caused by D-gal through the inhibition of both cellular senescence and oxidative stress. The study provides further evidence for drug development of MAT for prevention or treatment of the aging-associated disorder.

## 1. Introduction

As the population increases and lifespan is extended, the aging population becomes large and aging-related diseases have brought great attention worldwide. Aging is a biological process featured as a progressive degeneration of physiological functions which results in an increase in morbidity and death rate. Aging is one of the major factors of brain decline involved in gradual learning and memory loss, cognitive disorders, and dementia, like Alzheimer's disease [1, 2]. Moreover, fatigue is easily felt in both men and women as aging; the fatigue prevalence reached its highest level when the individuals reached the age of 90 years for both men and women [3]. Fatigue is associated with immune disorder and dysfunction of the antioxidant defense system, and so on [4, 5]. Harman proposed the free radical theory in 1956, and since then, numerous studies have proved that reactive oxygen species (ROS) play an essential role in aging [6–8]. High levels of ROS could induce oxidative stress and destroy the structure of DNA, phospholipids, and proteins, finally resulting in cellular and tissue injury [9]. This is a critical mechanism for ROS-induced aging. Meanwhile, mammalian cells have an antioxidant system able to scavenge high levels of ROS. Total superoxide dismutase (T-SOD), catalase (CAT), and vitamin E (VE) are the critical members of this antioxidant system [10].

The previous study has shown that chronic exposure to D-galactose (D-gal) caused similar symptoms such as natural aging [11]. Overload of D-gal could accelerate the production of ROS and lead to oxidative stress [12]. Brain and liver dysfunction is closely relative to aging, and it is susceptible to D-gal caused by oxidative injury [13–15]. Furthermore, it has shown that the number of senescent cells and apoptosis in the liver and brain increased in D-gal-induced aging mice [16, 17]. Recent studies have shown that senescent cells accumulated in various aging and diseased tissues [18]. Cellular senescence is associated with age-related phenotypes causally, and the decrease in senescent cells can retard tissue dysfunction and extend healthspan [19]. Hence, a D-gal-induced aging model has been widely used for pharmacological studies of antiaging agents.

MAT, the major alkaloids extracted from traditional Chinese medicine ***Sophora flavescens***, has numerous pharmacological activities, such as antitumor, antidiabetes, and cardioprotective effects [20–23]. MAT not only attenuates focal cerebral ischemic injury [24] but also protects against MTPT-induced Parkinson's disease via antioxidative stress [25]. However, whether MAT protects against aging induced by D-gal and the underlying mechanisms have not been addressed yet. In the present study, we employed a D-gal-induced aging mouse model to investigate the protective effect of MAT. We found that D-gal treatment induced noticeably aging-related changes, including cognitive impairment and memory deficits; decrease in swimming time in the weight-loaded swimming test and thymus coefficient; increase in senescence-associated *β*-galactosidase (SA-*β*-gal) activity; *p16*, *p19*, *p21*, interleukin- (*IL-*) *1β* and *IL-6* gene expression; and oxidative stress in the liver, spleen, and brain. MAT's effect on memory deficit and endurance decline as well as the cellular senescence and oxidative stress levels were detected.

## 2. Materials and Methods

### 2.1. Animals and Drug Administration

Male ICR mice (8-week-old, 30-35 g) were purchased from Shanxi Medical University (Shanxi, China). Mice were housed 5 per cage, allowed to access food and water freely, and maintained in constant temperature (23 ± 1°C) and humidity (55 ± 5%) under a 12 h light/dark cycle (lights on 08 : 00 to 20 : 00 h). The investigation conformed to the International Guiding Principles for Biomedical Research Involving Animals (1985). The mice were used in each experiment to minimize both the number of animals used and their suffering. All experimental procedures were conducted and performed as the policies for animal care and use encompass regulations approved by the Institutional Animal Care and Use Committee of Shanxi Agricultural University.

The mice were randomly divided into five groups (9 mice in one group): control group (2% ethanol in saline + saline), D-gal group (2% ethanol in saline + 150 mg/kg D-gal), VE group (VE 100 mg/kg + 150 mg/kg D-gal), MAT low-dose group (MAT 2 mg/kg + 150 mg/kg D-gal), and MAT high-dose group (MAT 10 mg/kg + 150 mg/kg D-gal). The mice were treated with D-gal at a dose of 150 mg/kg for 6 weeks (s.c.). At the fifth week, the mice were given MAT or solvent (2% ethanol in saline, oral) for 4 weeks before the behavior test.

### 2.2. Drugs and Chemicals

MAT and VE were purchased from Aladdin Reagent Company (Shanghai, China); D-gal was purchased from Beijing Solarbio Science & Technology Company (Beijing, China). All other materials were of the highest grade available.

### 2.3. Novel Object Recognition (NOR) Task

Learning and memory ability was detected by the NOR task as described previously [26]. The dimensions of the apparatus are 40 cm × 25 cm × 20 cm (length × width × height). Mice had been put into the apparatus for 5 min without objects for two days. The apparatus was cleaned using 70% ethanol to eliminate the residual odor until dry. On the third day, the mice were tested by comparing two sessions: the training and test sessions. In the training session, the mice facing the wall were placed into the apparatus containing two identical objects and allowed to explore for 5 min. The objects were placed in opposite adjacent corners, 5 cm away from the walls. The test session was performed 60 min after the training session, with one object instead of a novel one. Exploration was defined as the condition for which the mice directed the head or nose to the object no more than 1 cm or touched the object. However, if the mouse sited on the object, it was not considered as exploration. The discrimination ratio of each mouse is defined as TN/(TN + TF) ratio (TF = exploring time on the familiar object; TN = exploring time on the novel object).

### 2.4. Spontaneous Alternation Behavior Y-Maze Test

The Y-maze test was performed after NOR according to the previously described method [27]. The Y-maze contained three dark, polyvinyl plastic arms with 120° angle between all arms, which were 30 cm long and 5 cm wide with 12 cm high walls. Mice were initially placed at the end of one arm and allowed to explore in the Y maze freely. The number of arm entries and the sequence of arm visits were recorded manually for each mouse for 8 min. Then, the mouse was removed from the Y-maze to its house, and the Y-maze was cleaned with 70% ethanol to remove the odor until dry. The mouse consecutively entering into three different arms was defined as an actual alteration, i.e., BCA, ABC, or CAB, but not ABB. Moreover, the percent of alteration was calculated as the number of actual alteration/total number of entering × 100.

### 2.5. Weight-Loaded Swimming Test

The weight-loaded swimming test was performed as described previously [28]. Briefly, the dimensions of the tank are 30 cm × 35 cm × 60 cm (length × width × height) and filled with water (23°C ± 1°C). The mice were loaded aluminum (5% of their body weight) attached to the tail, and the fur of the mice was dripping wet with water. Then, the mice were placed into the tank individually. The time at which the animals were unable to bail out the water surface for 5 seconds was recorded.

### 2.6. Body Weight and Organ Coefficient Analysis

The mice were weighed every week. After the weight-loaded swimming test, the mice were sacrificed, and the weights of the organ were weighted by an accurate electronic balance. The tissue was stored at −20°C until used for the biochemical studies. The organ coefficient was calculated using the following equation: organ index (g/100 g) = organ weight/body weight × 100.

### 2.7. Morphological and Histological Analyses

The liver, spleen, and brain tissues were excised, fixed, and embedded in 4% paraformaldehyde. The sections (5 *μ*m) were stained with haematoxylin and eosin (HE) (Beijing Solarbio Science & Technology Company, Beijing, China). Briefly, the sections were deparaffinized with 100% xylene and rehydrated with gradient alcohol (100% ethanol, 90% ethanol, 80% ethanol, and 70% ethanol) and distilled water. Then, the sections were washed in distilled water for three times and stained with haematoxylin for 10 minutes, differentiated in the differentiation liquid, and then washed with running tap water for 15 min. The nucleus was counterstained with eosin for 1 min and dehydrated. The pathological changes were observed under an optical microscope (Olympus, Japan). The photograph was analyzed by ImageJ. The related nuclear size was compared to the control group; the percentage of the white pulp of the spleen = white pulp area/total area.

### 2.8. Senescence-Associated *β*-Galactosidase (SA-*β*-Gal) Staining

The percentage of SA-*β*-gal-positive cells was determined using a senescence *β*-galactosidase staining kit (Beyotime Institute of Biotechnology, Haimen, China). Briefly, the sections (10 *μ*m) were washed with PBS three times and then incubated for 48 hours in SA-*β*-gal staining solution at 37°C. Then, the samples were washed with PBS three times and coverslipped for direct imaging with a microscope (Olympus, Japan).

### 2.9. Biochemical Analysis

Before detection, the tissues were rapidly homogenized in ice-cold saline (9 times of tissue weight) with a homogenizer. The homogenates were centrifuged at 4500 g at 4°C for 15 min, and the supernatant was collected for determination of acetylcholinesterase (AChE), total antioxidant capacity (T-AOC), SOD, CAT activities, and malondialdehyde (MDA) level. Protein concentrations were determined using a protein assay kit (Bio-Rad Laboratories, Hercules, USA). The total antioxidant capacity, total superoxide dismutase, CAT, AChE, and MDA detection kits (Nanjing Jiancheng Bioengineering Institute, Nanjing, China) were used to determine the T-AOC, SOD, CAT, and AChE activities and MDA level according to the protocols. The absorbance was measured at 405, 520, 532, 412, or 550 nm wavelengths at room temperature. The activities of T-AOC, SOD, CAT, and AChE were expressed in U/mg protein, and the MDA level was shown in nmol/mg protein, respectively.

### 2.10. RNA Extraction and Quantitative PCR

Total RNA was isolated from the liver and hippocampus of mice with TRIzol reagent (Invitrogen, Carlsbad, CA). NanoDrop 1000 (Thermo, Wilmington, USA) was used to quantify the amount of total RNA. RNA at the amount of 2.5 *μ*g was reverse-transcribed and analyzed by quantitative PCR with SYBR Premix EX Taq™ (Takara, Dalian, China). All real-time reactions were performed on the ABI7500 Multicolor Real-Time PCR detection system (Thermo, Wilmington, USA). A three-step PCR procedure of 20 s at 95°C, 20 s at 55°C, and 20 s at 68°C was applied for 40 cycles. The primer sequences are shown in Table 1. All results were normalized to 18S RNA levels, and the data were analyzed using the 2^−ΔΔCT^ method.

### 2.11. Statistical Analyses

The results of the behavior tests, organ index, enzyme activities, MDA level, and related gene expression are expressed as mean ± SEM. Data were analyzed by one-way analysis of variance (ANOVA) followed by Turkey's test for post hoc analysis. For novel object recognition, statistical evaluation was performed by a paired sample *t*-test. Statistical significance was set at *p* < 0.05.

## 3. Results

### 3.1. Effect of MAT on D-Gal-Induced Cognitive Impairment in the Object Recognition Task

**Figure 1:**
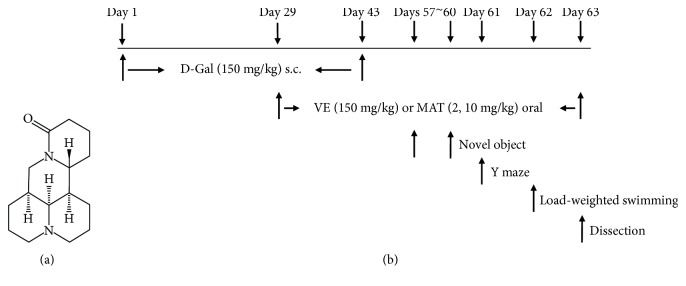
(a) The chemical structure of MAT and (b) experimental protocol in this study.

**Figure 2:**
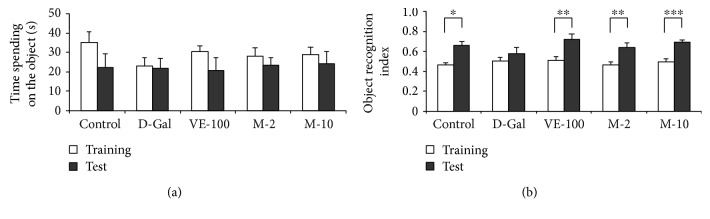
MAT effects on D-gal-induced memory impairment in the novel object recognition test. MAT (2 and 10 mg/kg, oral) was administrated to D-gal-treated mice. D-gal was injected at a dose of 150 mg/kg for six weeks (s.c.). (a) Time spent on the object in seconds and (b) object recognition index were observed. Results are presented as mean ± SEM. *n* = 9, ^∗^ *p* < 0.05, ^∗∗^ *p* < 0.01, and ^∗∗∗^ *p* < 0.001, differences between recognition index in the training and test session. Control received 2% ethanol in saline + saline, D-gal received 2% ethanol in saline + 150 mg/kg D-gal, VE-100 received 100 mg/kg VE + 150 mg/kg D-gal, M-2 received 2 mg/kg matrine + 150 mg/kg D-gal, and M-10 received 10 mg/kg matrine + 150 mg/kg D-gal.

The chemical structure of MAT and experimental protocol in this study are shown in Figure 1. The influence of pretreatment with MAT on D-gal-induced memory impairment was initially investigated in the NOR task. VE was used as a standard positive control. Usually, the mice spent more time on the novel object compared to the familiar one. So, compared with the training session, there was a significant increase in the object recognition index in the test session in the control group (*p* < 0.05). D-gal caused a decrease in the difference between training and test for the object recognition index with the comparison of the control group, whereas pretreatment of VE (100 mg/kg) and MAT (2 and 10 mg/kg, oral) abolished the partial amnesic effect of D-gal (Figure 2(b)) (*p* < 0.01, *p* < 0.01, and *p* < 0.001). As observed, long-term treatment with D-gal could induce recognition deficit in the NOR test, and this was improved by treatment with VE or MAT. No significant differences were seen in total time spent on the object in all groups (Figure 2(a)). These results suggest that MAT could improve the reference memory impairment caused by D-gal.

### 3.2. Effect of MAT on D-Gal-Induced Learning and Memory Decline in the Y-Maze

**Figure 3:**
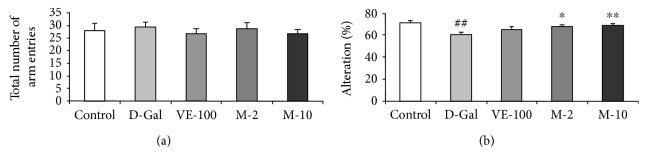
MAT effects on the Y-maze test. The mice were put into the Y-maze for 8 min one by one. (a) The total number of arm entries and (b) alternations were measured. The data are expressed as mean ± SEM. *n* = 9, ^##^ *p* < 0.01 vs. vehicle control group and ^∗^ *p* < 0.05 and ^∗∗^ *p* < 0.01 vs. D-gal-treated group.

The impact of MAT on working memory was investigated in the Y-maze test. D-gal significantly decreased spontaneous alternation relative to the control group (Figure 3(b)) (*p* < 0.01). Moreover, treatment with MAT (2 and 10 mg/kg) significantly reversed the spontaneous alternation decline caused by D-gal (Figure 3(b)) (*p* < 0.05, *p* < 0.01). However, VE at 100 mg/kg did not take effect on alternation decline compared with the D-Gal-treated group (Figure 3(b)). There were no significant differences in arm entries among all the groups (Figure 3(a)). It means that MAT could improve the working memory and short memory but did not affect the locomotor activity of the D-Gal induced model mice.

### 3.3. Effect of MAT on the Weight-Loaded Forced Swimming Test

**Figure 4:**
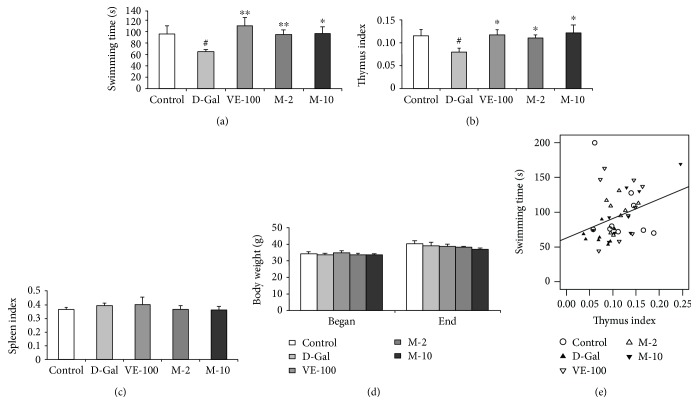
Effect of MAT on weight-loaded swimming test, thymus and spleen coefficients, body weight in the D-gal-induced aging mice, and Pearson's correlation between the swimming time versus thymus coefficients. (a) The weight-loaded swimming test: mice were loaded aluminum (5% of their body weight) attached to the tail, and the swimming time was recorded in seconds. (b) The thymus and (c) spleen coefficients: the animals were decapitated after the weight-loaded swimming test, and thymus coefficients were analyzed. (d) The body weight changes between the start of experiment and the end of the experiment. The data are expressed as mean ± SEM. *n* = 9, ^#^ *p* < 0.05 vs. vehicle control group and ^∗^ *p* < 0.05, ^∗∗^ *p* < 0.01, and ^∗∗∗^ *p* < 0.001 vs. D-gal-treated group. (e) Pearson's correlation between swimming time versus thymus coefficients was determined. Control group (saline + 2% ethanol in saline, hollow circle), D-gal group (D-gal 150 mg/kg + 2% ethanol in saline, filled triangle), VE-100 (D-gal 150 mg/kg + VE 100 mg/kg, inverted hollow triangle), M-2 (D-gal 150 mg/kg + matrine 2 mg/kg, hollow triangle), and M-10 (D-gal 150 mg/kg + matrine 10 mg/kg, inverted filled triangle), *n* = 45.

The antifatigue impact of MAT was determined in mice by measuring the swimming duration in weight-loaded forced swimming. D-gal-treated mice decreased swimming time when compared with the control group (*p* < 0.05) (Figure 4(a)). However, treatment with VE and MAT (2 and 10 mg/kg) significantly reversed the swimming time decline caused by D-gal (Figure 4(a)) (*p* < 0.01, *p* < 0.01, and *p* < 0.05). It means that MAT could improve the physical power of aging mice which is induced by D-gal.

### 3.4. Effect of MAT on Body Weight and Organ Indexes

In mammals, retrogression of immune organs with aging results in the decline of the immune organ coefficients, such as thymus coefficient, which is usually used as a hallmark to evaluate the success of the aging animal model. In the present study, 6 weeks of D-gal treatment induced significant decreases in thymus coefficient (*p* < 0.05) (Figure 4(b)). VE and MAT supplementation dramatically antagonized such changes induced by D-gal treatment (*p* < 0.05 for all groups), suggesting the effective immune organ protection of MAT and VE. However, there was no notable difference in body weight and spleen index among groups (Figures 4(c) and 4(d)).

More importantly, when the linear regression was determined, there was a significant positive correlation between the swimming time versus thymus coefficient (*n* = 45, *r* = 0.323, *p* < 0.05) (Figure 4(e)).

### 3.5. Effect of MAT on Liver, Spleen, and Brain Histopathological Alterations

**Figure 5:**
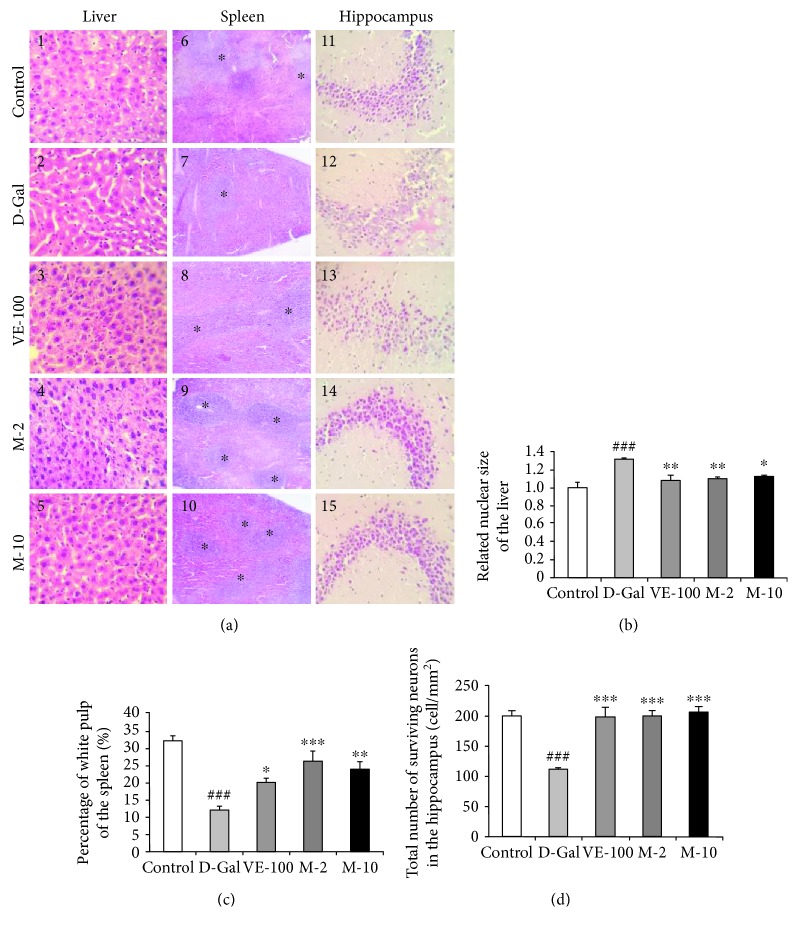
Effect of MAT on liver, spleen, and brain histopathological alterations. (a) The histopathological alterations of the liver, spleen, and brain (HE); the magnification of the liver and brain is 400 times, and that of spleen is 100 times. (b) The related nuclear size of the liver, (c) spleen white pulp proportion, and (d) surviving neurons of CA3 area of the hippocampus. The data are expressed as mean ± SEM, *n* = 6. ^##^ *p* < 0.01 and ^###^ *p* < 0.001 indicate significant difference compared with the control group; ^∗^ *p* < 0.05, ^∗∗^ *p* < 0.01, and ^∗∗∗^ *p* < 0.001 indicate significant difference compared with the D-gal-treated control group.

The histological features of the liver sections by HE stained are shown in Figure 5(a), 1~5. The D-gal-treated group exhibited a dramatic increase in the related nuclear size (*p* < 0.001), and VE and MAT at 2 and 10 mg/kg significantly reversed such changes (*p* < 0.01, *p* < 0.01, and *p* < 0.05). D-gal obviously decreased the percentage of the white pulp of the spleen (Figure 5(a), 6, *p* < 0.001), and VE and MAT markedly increased the percentage of white pulp area decline induced by D-gal (Figure 5(a), 7~10, *p* < 0.05, *p* < 0.001, and *p* < 0.01). Changes in histopathology features of the CA3 region of the hippocampus are shown in Figure 5(a), 11~15. It was demonstrated that the structure of the cell was damaged severely, such as cell apoptosis and necrosis in the D-gal treated group. In contrast, MAT-treated mice showed a reduction in damaged cells. A decline in the number of surviving neurons in the CA3 region was observed in the D-gal group compared with the control (*p* < 0.001). Treatment with VE and MAT produced an obvious improvement on such changes (*p* < 0.001 for all groups).

### 3.6. MAT Effect on the SA-*β*-Gal Activity of the Liver, Spleen, and Brain

**Figure 6:**
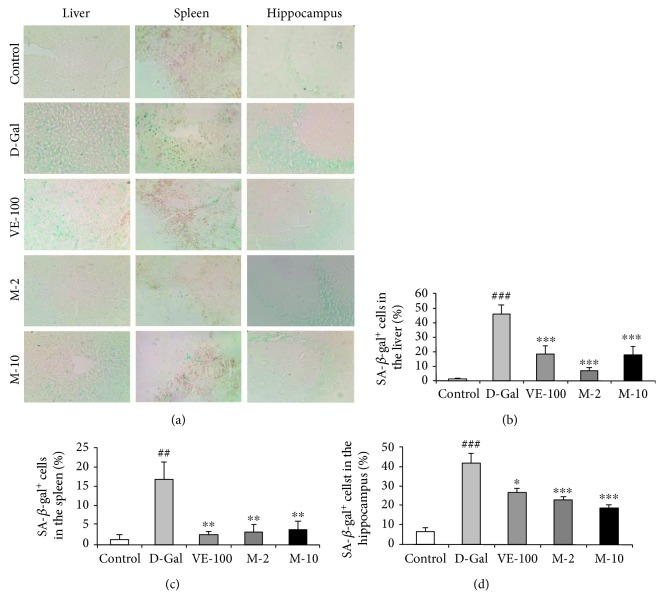
MAT attenuated the increased activity of SA-*β*-Gal in liver, spleen, and brain of D-gal-induced aging mice. (a) Representative image of SA-*β*-gal staining in each group was captured (magnification 400x). (b–d) Quantification of the percentage of SA-*β*-gal-positive cell number in the liver, spleen, and hippocampus. Blue staining means SA-*β*-gal-positive cells. The data are expressed as mean ± SEM, *n* = 6. ^##^ *p* < 0.01 and ^###^ *p* < 0.001 indicate significant difference compared with the control group; ^∗^ *p* < 0.05, ^∗∗^ *p* < 0.01, and ^∗∗∗^ *p* < 0.001 indicate significant difference compared with the D-gal-treated control group.

In order to confirm the protection of MAT, we chose the liver, spleen, and brain as the representative organs to detect the most widely recognized aging biomarker, SA-*β*-gal activity. D-gal treatment greatly increased the percentage of SA-*β*-gal-positive cells in the liver, spleen, and brain (represented by blue staining in the images, Figure 6) compared with the control group (*p* < 0.001, *p* < 0.01, and *p* < 0.001). These confirmed the aging changes of the liver, spleen, and brain. Moreover, supplementation with VE and MAT showed effective inhibition of the percentage of SA-*β*-gal-positive cells in the liver (*p* < 0.001 for all groups), spleen (*p* < 0.01 for all groups), and brain (*p* < 0.05, *p* < 0.001, and *p* < 0.001), further confirming the antiaging effect of MAT. These results show that MAT effectively attenuates D-gal-induced aging in mice.

More importantly, linear regression was determined which showed that there was a significant positive correlation between the related nuclear size of the liver versus the percentage of SA-*β*-gal-positive cell number in the liver (Figure S1A, *r* = 0.551, *p* < 0.01) and a negative correlation between the percentage of white pulp of the spleen versus the percentage of SA-*β*-gal-positive cell number in the spleen (Figure S1B, *r* = −0.622, *p* < 0.001) and between the survival cells in the hippocampus versus the percentage of SA-*β*-gal-positive cell number in the hippocampus (Figure S1C, *r* = −0.622, *p* < 0.001).

### 3.7. Antioxidative Effect of MAT in Liver, Plasma, and Brain of D-Gal-Induced Aging Mice

**Figure 7:**
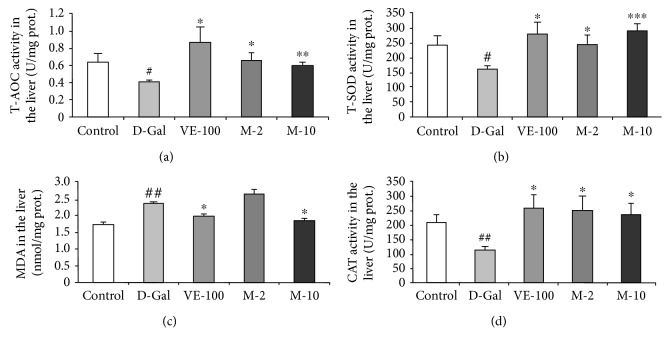
Effect of MAT on T-AOC, T-SOD, MDA, and CAT activities in the liver of D-gal-induced mice in vivo. (a) Changes in T-AOC, (b) T-SOD activity, (c) MDA level, and (d) CAT activity in the liver. Each value represents the mean ± SEM of 6 mice. ^#^ *p* < 0.05 and ^##^ *p* < 0.01 indicate significant difference compared with the control group; ^∗^ *p* < 0.05, ^∗∗^ *p* < 0.01, and ^∗∗∗^ *p* < 0.001 indicate significant difference compared with the D-gal-treated control group.

**Figure 8:**
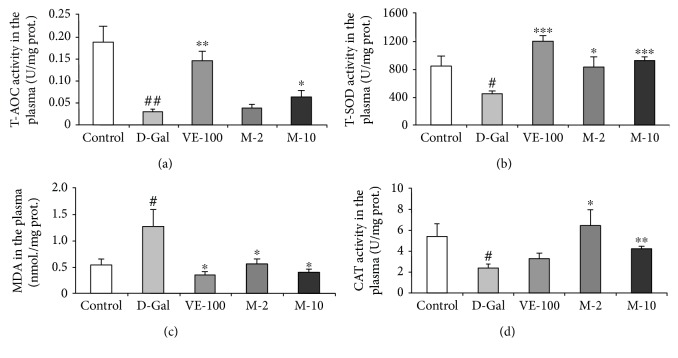
Effect of MAT on T-AOC, T-SOD, and MDA levels and CAT activity in the plasma of D-gal-induced mice in vivo. (a) Changes in T-AOC, (b) T-SOD activity, (c) MDA level, and (d) CAT activity in the plasma. Each value represents mean ± SEM of 6 mice. ^#^ *p* < 0.05 and ^##^ *p* < 0.01 indicate significant difference compared with the control group; ^∗^ *p* < 0.05, ^∗∗^ *p* < 0.01, and ^∗∗∗^ *p* < 0.001 indicate significant difference compared with the D-gal-treated control group.

**Figure 9:**
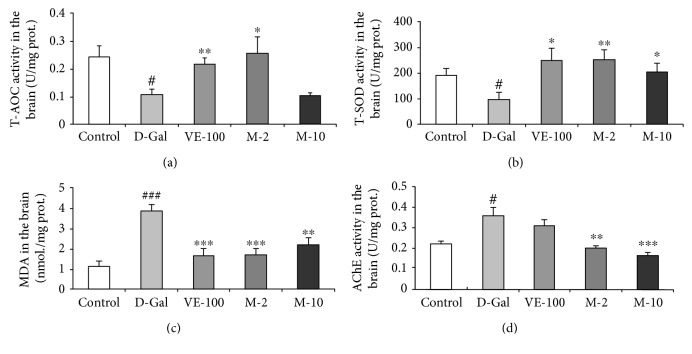
Effect of MAT on T-AOC, T-SOD, MDA, and AChE activities in the brain. (a) Changes in T-AOC, (b) T-SOD activity, (c) MDA level, and (d) AChE activity in the brain. Each value represents mean ± SEM of 6 mice. ^#^ *p* < 0.05 and ^###^ *p* < 0.001 indicates significant difference compared with the control group; ^∗^ *p* < 0.05, ^∗∗^ *p* < 0.01, and ^∗∗∗^ *p* < 0.001 indicate significant difference compared with the D-gal-treated control group.

Besides that, we also examined the antioxidative effect of MAT on D-gal-induced aging mice. T-AOC was analyzed firstly in the liver, plasma, and brain. It was shown that D-gal significantly decreased T-AOC in all three tissues compared with the control group (*p* < 0.05, *p* < 0.01, and *p* < 0.05), and VE and MAT increased T-AOC compared with the D-gal-treated group in the liver (*p* < 0.05, *p* < 0.05, and *p* < 0.01), plasma (*p* < 0.01, *p* < 0.05), and brain (*p* < 0.01, *p* < 0.05) (Figures 7(a) 8(a), and 9(a)). Then, the T-SOD and CAT activities and MDA level in the liver, plasma, and brain were determined by assay kit. The results showed that relative to the control group, the activity of T-SOD decreased in model mice (Figures 7(b) 8(b), and 9(b), *p* < 0.05 for all three tissues). Moreover, compared with the model mice, T-SOD increased in the liver (*p* < 0.05, *p* < 0.05, and *p* < 0.001), plasma (*p* < 0.001, *p* < 0.05, and *p* < 0.001), and brain (*p* < 0.05, *p* < 0.01, and *p* < 0.05) of the VE- and MAT-treated group at different concentrations (Figures 7(b) 8(b), and 9(b)). Meanwhile, the MDA level increased in the D-gal-treated group in the liver (*p* < 0.01), plasma (*p* < 0.05), and brain (*p* < 0.001), whereas MDA decreased in VE at 100 mg/kg and MAT at the 2 and 10 mg/kg-treated group in the plasma (*p* < 0.05 for all groups) and brain (*p* < 0.001, *p* < 0.001, and *p* < 0.01), and MDA decreased in the liver upon treatment with VE at 100 mg/kg and MAT at 10 mg/kg (*p* < 0.05 for both groups) (Figures 7(c) 8(c) and 9(c)). The CAT activity reduced in the D-gal-treated group in the liver (*p* < 0.01) and plasma (*p* < 0.05), whereas it was significantly higher than the D-gal-treated group in the liver of the VE- and MAT-treated groups (*p* < 0.05 for all groups) and in the plasma of the MAT-treated groups (*p* < 0.05, *p* < 0.01) (Figures 7(d) and 8(d)). However, there were no significant differences in CAT activity among all groups in the brain (data not shown). Moreover, we detected the AChE activity in the brain. D-gal obviously increased the AChE level in the brain (*p* < 0.05) relative to the control group, and MAT at both doses inhibited the AChE increase induced by D-gal (*p* < 0.01, *p* < 0.001); however, VE did not affect the AChE activity increase elicited by D-gal treatment (Figure 9(d)).

### 3.8. MAT Effect on p16, p19, p21, IL-1*β*, and IL-6 Gene Expression in the Liver and Hippocampus of D-Gal-Induced Aging Mice

**Figure 10:**
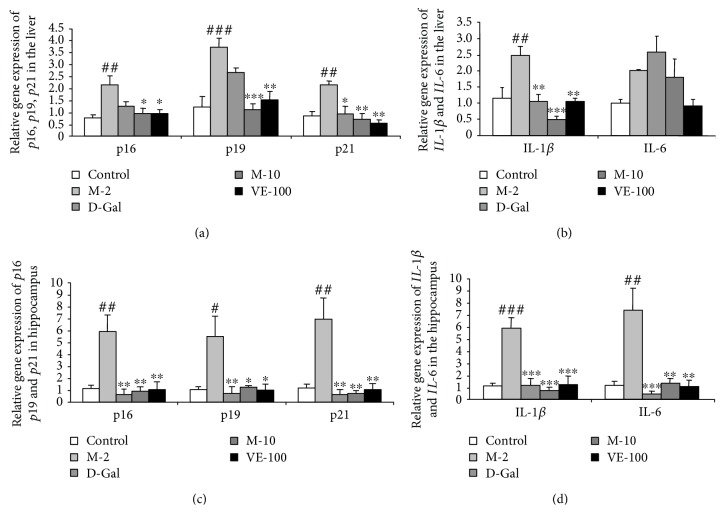
Effect of MAT on *p16*, *p19*, *p21*, *IL-1β*, and *IL-6* gene expression in the liver (a, b) and hippocampus (c, d) of D-gal-induced aging mice. Each value represents mean ± SEM of 4 mice. ^#^ *p* < 0.05, ^##^ *p* < 0.01, and ^###^ *p* < 0.001 indicate significant difference compared with the control group; ^∗^ *p* < 0.05, ^∗∗^ *p* < 0.01, and ^∗∗∗^ *p* < 0.001 indicate significant difference compared with the D-gal-treated control group.

To confirm the effect of MAT on the prevention of cellular senescence, we detected the *p16*, *p19*, and *p21* gene expression in the liver and hippocampus. The results showed that the D-gal treated group significantly increased the *p16*, *p19*, and *p21* gene expression in the liver (*p* < 0.01, *p* < 0.001, and *p* < 0.01) and hippocampus (*p* < 0.01, *p* < 0.05, and *p* < 0.01) compared with the control group; VE (100 mg/kg) apparently decreased *p21* gene expression of the liver (*p* < 0.05) or MAT (2, 10 mg/kg) and inhibited *p16*, *p19*, and *p21* gene expression obviously compared with the D-gal-treated group in the liver (*p* < 0.05, *p* < 0.05), (*p* < 0.001, *p* < 0.01), (*p* < 0.01, *p* < 0.01) (Figure 10(a)). Meanwhile, *p16*, *p19*, and *p21* gene expression decreased significantly in the hippocampus after treatment with VE (100 mg/kg) and MAT (2, 10 mg/kg) (*p* < 0.01, *p* < 0.01, and *p* < 0.01), (*p* < 0.01, *p* < 0.05, and *p* < 0.05), (*p* < 0.01, *p* < 0.01, and *p* < 0.01) (Figure 10(c)). Then, we detected the classic components of SASP and the *IL-1β* and *IL-6* expression. Related to the control group, the D-gal-treated group significantly increased the *IL-1β* and *IL-6* gene expression in the hippocampus (*p* < 0.001, *p* < 0.01); meanwhile, it notably increased *IL-1β* gene expression in the liver (*p* < 0.01). VE and MAT at doses of 2 and 10 mg/kg inhibited the *IL-1β* and *IL-6* gene expression compared with the D-gal-treated group in the hippocampus (*p* < 0.001 for all groups) (*p* < 0.001, *p* < 0.01, and *p* < 0.01). Related to the D-gal group, the *IL-1β* gene expression of the liver decreased obviously in the VE and MAT groups (*p* < 0.01, *p* < 0.001, and *p* < 0.01) (Figures 10(b) and 10(d)).

## 4. Discussion

Aging is an inevitable process which can affect many functions, such as immune, learning and memory, and exercise endurance. In recent years, the discovery of antiaging active substances has become a hot topic. Chinese Traditional Medicine is a good source for the discovery of active substances. MAT is an alkaloid found in plants of the *Sophora* genus, which has been used for some treatments, including antidiabetic, antitumor, and isoproterenol-induced heart disease [20–23]. The present study was designed to assess the antiaging activity of MAT. The learning and memory improvement of MAT was investigated using the object recognition and Y-maze tests, and antifatigue effect of MAT was detected by the weight-loaded forced swimming test. We also examined whether the antiaging effect of MAT is involved in inhibition of cellular senescence and oxidative stress by analysis of HE and SA-*β*-gal staining in the liver, spleen, and brain, evaluating the T-AOC, T-SOD, CAT activities and MDA level in the liver, plasma, and brain and detecting related gene expressions of *p16*, *p19*, *p21*, *IL-1β*, and *IL-6* in the liver and hippocampus.

Previous studies suggested that MAT had neuroprotective effects: MAT alleviated MPTP-induced Parkinson's disease and early brain injury after experimental subarachnoid hemorrhage [25, 29]. However, the impact of MAT on D-gal-induced learning and memory decline has not been explored. Aging is one of the major factors of brain decline involved in gradual learning and memory loss, cognitive disorders, and dementia, like Alzheimer's disease [1, 2]. We used the NOR and Y-maze tasks to determine the influence of MAT on cognitive impairment and memory deficits. The results showed that D-gal decreased the object recognition index in the test session, and administration of MAT (2 and 10 mg/kg) was effective in preventing the reference memory impairment induced by D-gal (Figure 2(b)). Furthermore, MAT (2 and 10 mg/kg) significantly increased the alternation in mice and improved working memory decrease that is caused by D-gal (Figure 3(b)). There were no significant differences among the groups on the exploration time of the object and the number of arm entries; these data agree with the previous research work [30]. Lack of ACh is one of the pathophysiologies of AD, and AChE is the primary enzyme that inactivates ACh in the brain [31]. Changes in AChE activity are strongly correlated with learning decline and memory deficits [32]. In this study, D-gal significantly increased the activity of AChE in brain tissue of mice. In contrast, the results showed that MAT markedly reversed the AChE activity. HE and SA-*β*-gal staining implied that MAT might improve learning and memory ability by maintaining the shape and nuclear integrity of hippocampus CA3 neurons, decreasing the aging cells in the hippocampus in mice. These data are consistent with the results of behavior tests.

People are more prone to fatigue as they get older; fatigue prevalence reached its highest level when the individuals reached age 90 years old for both men and women [3]. The antifatigue effect of MAT was detected by the weight-loaded forced swimming test. The results showed that MAT increased the swimming time decline induced by D-gal; these suggested that MAT possesses an antifatigue effect. Fatigue is associated with immune disorder [4], so the organ index of the thymus and spleen was analyzed. The thymus coefficient reduced in the D-gal-treated group, and it was improved by MAT. However, the spleen coefficient did not change among the groups (Figure 4(c)). It did not consist with the previous studies; the D-gal group received daily s.c. injection of D-gal at a dose of 150 mg/kg for ten weeks [17]. This may because the injection time of D-gal and the mouse strain were different from the report. Next, we detected the histopathological change and aging cells of the spleen. Even there were no significant changes in the spleen index. However, the white pulp proportion reduced in the D-gal group compared with the control, and MAT improved the situation; MAT inhibited the aging cells in the spleen relative to the D-gal group. Also, there was an apparent positive correlation between the swimming time and thymus index. These data mean that MAT produces the antifatigue effect through regulation of immune organs.

The histopathological section showed that MAT protected the aging situation of the liver, spleen, and brain. The related nuclear size of the liver remarkably increased with aging [33], and D-gal apparently decreased spleen white pulp proportion [34] and the surviving neurons of the hippocampus [35] in the previous studies. Also, MAT significantly decreased the related nuclear size of the liver and increased the percentage of the white pulp area and surviving neurons relative to the D-gal group (Figure 5). Recent research work has demonstrated that senescent cells accumulated in various tissues of age and disease [18]. Cellular senescence is associated with age-related phenotypes causally, and decreasing senescent cells can retard tissue dysfunction and extend healthspan [19]. Senescent cell accumulation improved hepatic fat accumulation and liver steatosis, and the reduction in senescent cells by treatment with a combination of dasatinib and quercetin reduced hepatic steatosis globally [36]. The exposure to certain environmental toxins, such as paraquat, improved senescent cell accumulation in the aging brain, which can lead to neurodegeneration, and the elimination of senescent cells protected against paraquat-induced neuropathology [37]. These results suggest that senescent cells are an emerging target for aging-related diseases, and inhibition of senescent cells could delay aging. SA-*β*-gal accumulates in senescent cells and is widely used to determine senescence. The results indicate that MAT can protect against senescence of the liver, spleen, and brain. Oxidative stress is one of the major factors to contribute to cellular senescence, and a typical feature of senescence is a shift to a prooxidant redox state. Therefore, numerous researches have shown that treatment with antioxidants could delay the cellular senescence [38]. Moreover, SOD, CAT, and VE are the important members of this antioxidant system [10]. In our studies, MAT could significantly increase T-AOC and T-SOD in the liver, plasma, and brain and CAT in the plasma and liver. The influence of MAT on D-gal-induced MDA level increase was observed, and MAT significantly inhibited MDA levels in the plasma, liver, and brain.

*p19/p21* and *p16* are two major signal pathways during cellular senescence. An increasing expression level of *p21* and *p16* has been found in aged human and murine fibroblasts and melanocytes [39]. Accumulation of p16^Ink4a^-positive cells during adulthood shorten healthy lifespan and accelerate age-dependent changes in several organs [40]. Moreover, clearance of p16^Ink4a^-positive senescent cells delays aging-associated disorders [19]. The *p16* expression could be used as a biomarker of physiologic age [41]. The deletion of *p21* improved stem cell function and lifespan of mice with dysfunctional telomeres without accelerating cancer formation [42]. Oxidative stress caused DNA damage and telomere uncapping, which induced *p19/p21* activation and activated *p16* expression through p38-MAPK. *p16* and *p19/p21* activation promoted cellular senescence [39]. To confirm the effect of MAT on prevention of cellular senescence, we detected the *p16*, *p19*, and *p21* gene expression in the liver and hippocampus. The results showed that D-gal increased *p16*, *p19*, and *p21* gene expression in the liver and hippocampus as previously reported [16, 43, 44]. MAT significantly ameliorated such changes. Furthermore, we detected the classic components of SASP, *IL-1β*, and *IL-6* expression in the hippocampus and liver. The results showed that MAT obviously inhibited the increase in *IL-1β* and *IL-6* in the hippocampus and *IL-1β* expression in the liver that is caused by D-gal. These results are consistent with SA-*β*-gal staining. Our results suggest that the antiaging effects of MAT may be due to the inhibition of cellular senescence and oxidative stress.

## 5. Conclusion

In summary, the present research work provided evidence that MAT ameliorated the D-gal-induced cognitive impairment and memory deficits in the NOR and Y-maze tasks in mice, as well as fatigue in the weight-loaded forced swimming test. HE and SA-*β*-gal staining showed that MAT could protect liver, spleen, and brain aging. In addition, the potent antiaging effects of MAT may be partly linked to inhibition of cellular senescence and antioxidant activity in the liver, plasma, and brain. These suggest that MAT may be a candidate for the prevention or treatment of aging-relative disorders (Figure S2).

## Figures and Tables

**Table 1:** Forward and reverse primers for quantitative real-time PCR analysis.

Primer	Forward	Reverse
*18S*	5′-TAA CCC GTT GAA CCC CAT T-3′	5′-CCA TCC AAT CGG TAG TAG CG-3′
*p16*	5′-CTCAGCCCGCCTTTTTCTTC-3′	5′-CGCCTTCGCTCAGTTTCTCATG-3′
*p19*	5′-GTGGCTCTCGCTACTCTGTTG-3′	5′-ATAGTGGATACCGGTGGACCT-3′
*p21*	5′-ACTACCAGCTGTGGGGTGAG-3′	5′-TCGGACATCACCAGGATTGG-3′
*IL-6*	5′-CCCCAATTTCCAATGCTCTCC-3′	5′-CGCACTAGGTTTGCCGAGTA-3′
*IL-1β*	5′-GCCACCTTTTGACAGTGATGAG-3′	5′-GACAGCCCAGGTCAAAGGTT-3′

## Data Availability

The data used to support the findings of this study are included within the article and the supplementary information files.

## Abbreviations

AChE: Acetylcholinesterase
CAT: Catalase
D-gal: D-Galactose
HE: Haematoxylin and eosin
IL: Interleukin
MAT: Matrine
MDA: Malondialdehyde
NOR: Novel object recognition
ROS: Reactive oxygen species
SASP: Senescence-associated secretory phenotype
SA-*β*-gal: Senescence-associated *β*-galactosidase
T-AOC: Total antioxidant capacity
T-SOD: Total superoxide dismutase
VE: Vitamin E.

## Supplementary Materials

Supplementary Materials Figure S1: Pearson's correlation between histopathological alterations and cellular senescence of the liver, spleen, and hippocampus. Pearson's correlation (A) between the related nuclear size of the liver versus the percentage of SA-*β*-gal-positive cell number in the liver, (B) between the percentage of white pulp of the spleen versus the percentage of SA-*β*-gal-positive cell number in the spleen, and (C) between the survival cells in the hippocampus versus the percentage of SA-*β*-gal-positive cell number in the hippocampus was determined. Control group (saline + 2% ethanol in saline, hollow circle), D-gal group (D-gal 150 mg/kg + 2% ethanol in saline, filled triangle), VE-100 (D-gal 150 mg/kg + VE 100 mg/kg, inverted hollow triangle), M-2 (D-gal 150 mg/kg + matrine 2 mg/kg, hollow triangle), M-10 (D-gal 150 mg/kg + matrine 10 mg/kg, inverted filled triangle), *n* = 30. Figure S2: table of content. Overload D-gal could lead to oxidative stress, which is one of the major factors to contribute to cellular senescence. Cellular senescence is associated with age-related phenotypes causally, such as learning and memory deficits and exercise endurance decline. Moreover, the potent antiaging effects of MAT may be partly linked to the inhibition of oxidative stress and cellular senescence.
