# Supplementary figures and images for: Matrine Attenuates D-Galactose-Induced Aging-Related Behavior in Mice via Inhibition of Cellular Senescence and Oxidative Stress
Source: Oxid Med Cell Longev. 2018 Nov 27;2018:7108604. doi: 10.1155/2018/7108604 (PMC6288577; doi:10.1155/2018/7108604)

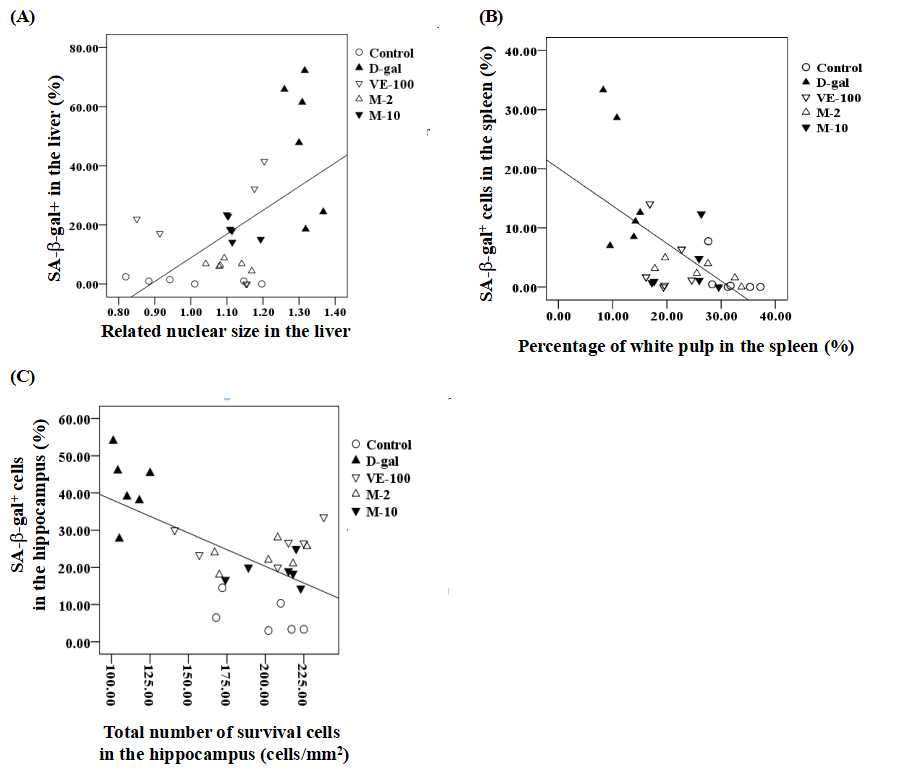


Figure S1


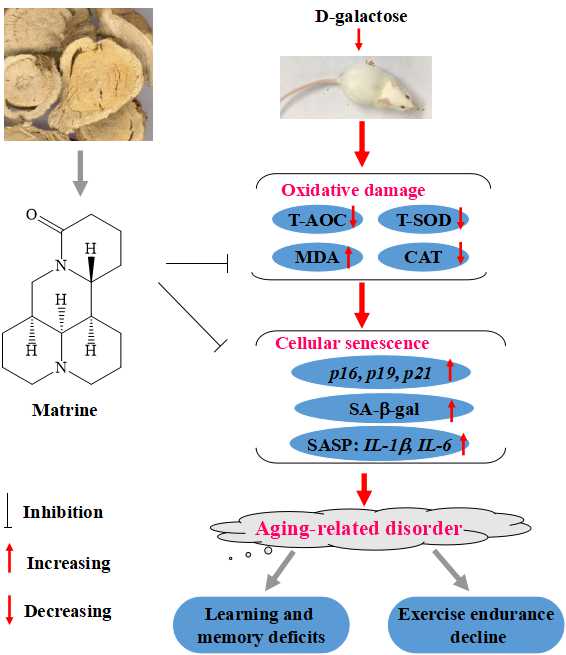


Figure S2

Supplement: Supplementary Materials — Figure S1: Pearson's correlation between histopathological alterations and cellular senescence of the liver, spleen, and hippocampus. Pearson's correlation (A) between the related nuclear size of the liver versus the percentage of SA-β-gal-positive cell number in the liver, (B) between the percentage of white pulp of the spleen versus the percentage of SA-β-gal-positive cell number in the spleen, and (C) between the survival cells in the hippocampus versus the percentage of SA-β-gal-positive cell number in the hippocampus was determined. Control group (saline + 2% ethanol in saline, hollow circle), D-gal group (D-gal 150 mg/kg + 2% ethanol in saline, filled triangle), VE-100 (D-gal 150 mg/kg + VE 100 mg/kg, inverted hollow triangle), M-2 (D-gal 150 mg/kg + matrine 2 mg/kg, hollow triangle), M-10 (D-gal 150 mg/kg + matrine 10 mg/kg, inverted filled triangle), n = 30. Figure S2: table of content. Overload D-gal could lead to oxidative stress, which is one of the major factors to contribute to cellular senescence. Cellular senescence is associated with age-related phenotypes causally, such as learning and memory deficits and exercise endurance decline. Moreover, the potent antiaging effects of MAT may be partly linked to the inhibition of oxidative stress and cellular senescence. [file 7108604.f1.docx]
